# Ultrasound findings of unplanned extraction of tunneled central venous catheters due to complications within subcutaneous tissue

**DOI:** 10.1007/s10396-025-01519-2

**Published:** 2025-03-03

**Authors:** Takahiro Hosokawa, Yumiko Sato, Yutaka Tanami, Eiji Oguma

**Affiliations:** Department of Radiology, Saitama Children’s Medical Center, 1-2 Shintoshin, Chuo-Ku, Saitama, Saitama 330-8777 Japan

**Keywords:** Hickman catheter, Broviac catheter, Tunneled central venous catheter, Infection, Fracture

## Abstract

**Purpose:**

Tunneled central venous catheters (CVC) are essential medical devices for pediatric patients facing extended treatment periods. This study aimed to demonstrate the usefulness of ultrasound in evaluating complications in subcutaneous tissue associated with unplanned extraction of tunneled CVC.

**Methods:**

Twenty-five patients who underwent ultrasound examination for suspected complications in the subcutaneous tissue associated with tunneled areas from CVC were included. The following patient characteristics and imaging findings were evaluated: infection in subcutaneous tissue, high echogenicity and hypoechoic effusion around the catheter within the subcutaneous tissue, and hyperechoic foci around the catheter. Patients with CVC were classified into two groups: those with and those without unplanned extraction of tunneled CVC. Fisher’s exact test was used to compare the two groups.

**Results:**

Nine patients had unplanned extraction of tunneled CVC. A significant difference was found in infection at tunneled areas (presence/absence in patients with vs. those without unplanned extraction = 7/2 vs. 2/14, P = 0.002), as well as in hypoechoic effusion around the catheter within the subcutaneous tissues (presence/absence in patients with vs. those without unplanned extraction = 9/0 vs. 3/13, P < 0.001). However, no significant differences were found in the presence or absence of high echogenicity (presence/absence in patients with vs. those without unplanned extraction = 7/2 vs. 6/10, P = 0.097) or hyperechoic foci around the catheter (presence/absence in patients with vs. those without unplanned extraction = 3/6 vs. 1/15, P = 0.116).

**Conclusion:**

The ultrasound findings were useful for determining the necessity of tunneled CVC extraction. These results will be helpful for improving management of pediatric patients with CVC.

## Introduction

Central venous catheters (CVC) are important medical devices for chemotherapy, fluid administration, antibiotic therapy, and parenteral nutrition. Approximately 8% of hospitalized patients require these devices [1, 2]. Improvements in medical technology have improved techniques for CVC insertion. For example, ultrasound can now be used to guide CVC insertion into the vascular lumen. This advance allows the devices to be more widely used in pediatric patients, particularly those who require extended treatment durations [3–6]. Several types of CVC, such as Hickman, Broviac, and port device catheters, have been implemented for long-term use and have been implanted in patients for several years [1].

Despite the advances in insertion techniques, complications such as infections, catheter fractures, thrombosis, and mispositioning of the catheter tip have been reported in cases of long-term CVC placement [2, 7–11]. A CVC uses subcutaneous tissues to prevent infection and enhance its utility [12, 13]. Ultrasound is useful for detecting thrombosis within the vascular lumen, but there are no reports that focus on CVC complications in the tunneled areas within the subcutaneous tissues [8, 11, 14–16]. General anesthesia is usually needed to re-insert a tunneled CVC in pediatric patients; therefore, physicians may be hesitant to retract the catheter.

This study aimed to demonstrate the usefulness of ultrasound in evaluating the necessity of tunneled CVC removal due to complications within subcutaneous tissues. The study also evaluated the association between sonographic findings and the time from symptom onset to CVC removal.

## Material and methods

### Ethical considerations

This retrospective study was conducted in accordance with the tenets of the Declaration of Helsinki and was approved by the ethics committee of our institution. The requirement for informed consent was waived owing to the retrospective nature of the study.

### Study population

We evaluated the electronic medical records of patients who were at our institution between May 2009 and July 2024. A total of 36 children aged < 16 years who underwent insertion of a tunneled Hickmann or Broviac catheter were evaluated, and ultrasound examinations were performed to assess suspected complications in tunneled areas in subcutaneous tissues from tunneled CVC. When physicians suspected complications from tunneled CVC, they ordered ultrasounds of the subcutaneous tissues based on the presence of fever, swelling, erythema, or pain at the tunneled areas.

The following patients were excluded:

1. Patients in whom a port device was used (n = 4), because their symptoms could have also been associated with port puncture or leakage at the puncture site.
2. Patients who had complications after removal of the catheter (n = 3).
3. Patients with a final diagnosis of infection at the catheter tip but not in the tunneled/subcutaneous area (n = 3).
4. Patients with suspected infection at the tunneled area, but in whom the bacterial culture from the subcutaneous tissue was negative (n = 1).

### Patient classification

Patients were classified as those with or without unplanned extraction of tunneled CVC. “Unplanned extraction of tunneled CVC” was defined as CVC extraction based on the clinical diagnosis of complications within the subcutaneous tissues associated with tunneled CVC. In this study, infection and catheter fracture at the tunneled area were the only complications that were observed.

Patient sex, age, time from catheter insertion to symptom onset, and time from symptom onset to catheter extraction were recorded.

### Complications associated with tunneled CVC within subcutaneous tissues by tunneled area as the final diagnosis

Complications within subcutaneous tissues associated with tunneled CVC were diagnosed based on the presence of a catheter fracture within the tunneled area or positive bacterial cultures in the subcutaneous tissue. “Without complications” was defined as the alleviation of symptoms without any medical/surgical intervention, or evidence of other causes such as infection at other sites.

### Ultrasound evaluation

#### Equipment

All postnatal sonograms were obtained using a linear transducer (9–15 MHz) or a convex transducer (2–9 MHz) (LOGIQ 7, E9, E10, and S8; GE Healthcare, Waukesha, WI, USA) by four radiologists with extensive experience in pediatric ultrasonography and radiology.

### Evaluation of ultrasound findings

**Fig. 1:**
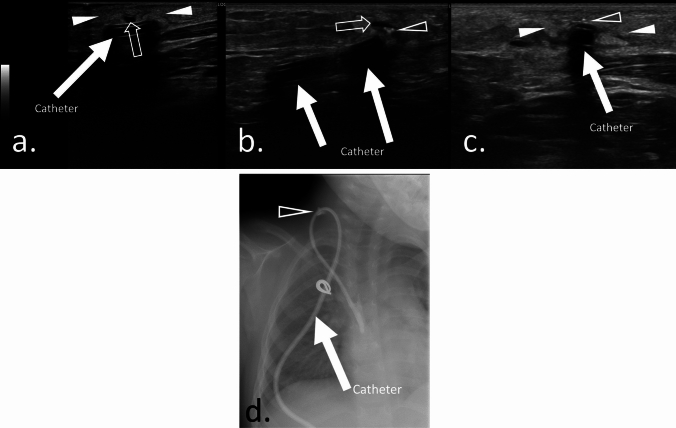
Case 1: Ultrasonographic findings in a 3.7-year-old girl with erythema and unplanned CVC extraction. The patient was diagnosed with complications within subcutaneous tissue due to catheter fracture at the tunneled area. The onset of symptoms was 45 days after catheter placement. The catheter was extracted 1 day after symptom onset. **a**. Ultrasonography showing a highly echoic area (solid arrowheads) around the catheter. Fluid collection around the catheter is also evident (vacant arrow). **b**. Ultrasonography showing fluid collection (vacant arrow) and highly echoic foci (vacant arrowhead) around the adjacent catheter. **c**. Hyperechoic foci (vacant arrowhead) and a highly echoic area (solid arrowheads) adjacent to the catheter were detected. **d**. Contrast study via catheter showing leakage of contrast medium (vacant arrowhead) from the catheter.

**Fig. 2:**
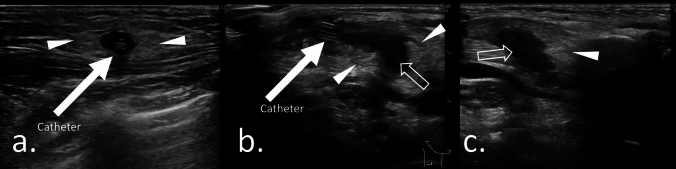
Case 8: Ultrasonographic findings in a 3.3-year-old boy with swelling, erythema, fever, and unplanned CVC extraction. The patient was diagnosed with complications within the subcutaneous tissue due to catheter infection at the tunneled area. The onset of symptoms was 296 days after catheter placement. The pathogen was *Staphylococcus aureus*. Catheter removal was performed 1 day after symptom onset. **a**. Ultrasonography showing a hyperechoic area (solid arrowheads) around the catheter. **b**. Ultrasonography indicated the presence of fluid collection (vacant arrow) around the catheter accompanying hyperechoic subcutaneous tissue (solid arrowheads). **c**. Ultrasonography showing fluid collection around the catheter (vacant arrow) without hyperechoic foci. A hyperechoic area is evident (solid arrowhead) within subcutaneous tissue

**Fig. 3:**
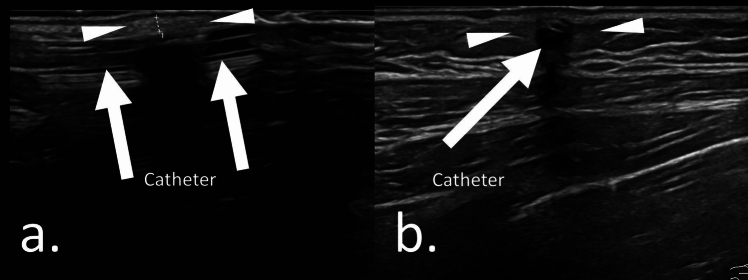
Case 19: Ultrasonographic findings in a 5.7-year-old boy with symptoms of erythema without unplanned CVC extraction. The patient was not diagnosed with complications associated with the tunneled catheter. The onset of symptoms was 112 days after catheter placement. Catheter removal was performed 154 days after symptom onset. **a**. Ultrasonography showing a hyperechoic area around the catheter (solid arrowheads) **b**. Ultrasonography does not show fluid collection around the catheter (solid arrowheads). Fluid collection and hyperechoic foci are absent.

The following imaging findings were evaluated [8, 14]:

1. High echogenicity around the catheter within subcutaneous tissues (Figs. 1–3).
2. Hypoechoic effusion around the catheter within subcutaneous tissues (Figs. 1 and 2).
3. Hyperechoic foci around the catheter (Fig. 1).

### Review process

All images were reviewed by two experienced pediatric radiologists using a 1600 × 1200 resolution picture archiving and communication system monitor (GE Healthcare, Waukesha, WI, USA). Discrepancies between the two evaluations were resolved through discussion. The reviewers were blinded to the patients’ surgical and physical data, as well as other imaging findings and the interpretation of the images.

### Statistical analysis

Data are presented as means ± standard deviation. Statistical significance was set at P < 0.05 (two-sided). All data were analyzed using a commercially available software program (SPSS version 24; IBM, Armonk, NY, USA).

### Patient characteristics

Fisher’s exact and Mann–Whitney U tests were used to compare pediatric patients with and without unplanned CVC extraction for the following variables: sex (male/female), age (years), presence or absence of diagnosis of tunneled area infection, time (days) from catheter insertion to symptom onset, and time (days) from symptom onset to catheter extraction.

### Image findings on ultrasound

Fisher’s exact test was used to compare the presence or absence of previous sonographic findings during ultrasound examination between the two groups.

Additionally, a Mann–Whitney U test was used to evaluate the association between the presence or absence of sonographic findings and the times from symptom onset to catheter removal.

## Results

### Patients

Patient characteristics are summarized in Table 1. A total of 25 patients (14 girls and 11 boys) were included in the study. The average age of the study participants was 6.2 ± 4.5 (range, 0.3–15.0) years, and the average time from catheter insertion to symptom onset was 98.0 ± 139.6 (range, 1–611) days.

**Table 1:** Patient characteristics and sonographic findings

No	Sex	Age (years)	Time from catheter insertion to symptom onset (days)	Symptom	Unplanned CVC extraction	Time from symptom onset to catheter removal (days)	Complication	Cause of complication	Pathogen of infection	Sonographic findings			Fig
										Hyperechoic area around catheter	Fluid collection	Hyperechoic foci	
1	F	3.7	45	Erythema	Present	1	Present	Fracture	–	Present	Present	Present	1
2	F	0.6	3	Swelling	Present	4	Present	Fracture	–	Absent	Present	Present	
3	F	10.9	19	Erythema	Present	0	Present	Infection	MRSA	Present	Present	Present	
4	M	9.8	22	Erythema	Present	10	Present	Infection	*Staphylococcus aureus*	Present	Present	Absent	
5	M	7.3	143	Erythema	Present	38	Present	Infection	*Staphylococcus aureus*	Present	Present	Absent	
6	F	7.0	611	Erythema	Present	47	Present	Infection	*Pseudomonas aeruginosa* *Serratia marcescens*	Present	Present	Absent	
7	M	6.5	105	Erythema	Present	13	Present	Infection	MRSA	Absent	Present	Absent	
8	M	3.3	296	Swelling, erythema, fever	Present	1	Present	Infection	*Staphylococcus aureus*	Present	Present	Absent	2
9	M	0.3	14	Erythema	Present	7	Present	Infection	*Enterococcus faecalis*	Present	Present	Absent	
10	F	3.2	67	Erythema	Absent	91	Present	Infection	MRSA	Present	Present	Present	
11	F	0.4	92	Erythema	Absent	86	Present	Infection	*Pseudomonas aeruginosa*	Present	Present	Absent	
12	M	15.0	159	Erythema	Absent	231	Absent	–	–	Absent	Present	Absent	
13	F	12.8	1	Pain	Absent	162	Absent	–	–	Absent	Absent	Absent	
14	F	12.3	8	Pain	Absent	273	Absent	–	–	Present	Absent	Absent	
15	F	12.2	8	Fever	Absent	218	Absent	–	–	Absent	Absent	Absent	
16	F	11.4	10	Pain	Absent	74	Absent	–	–	Present	Absent	Absent	
17	F	11.0	29	Erythema	Absent	287	Absent	–	–	Present	Absent	Absent	
18	F	7.4	389	Erythema	Absent	4	Absent	–	–	Absent	Absent	Absent	
19	M	5.7	112	Erythema	Absent	154	Absent	–	–	Present	Absent	Absent	3
20	M	5.6	16	Swelling	Absent	415	Absent	–	–	Absent	Absent	Absent	
21	M	2.6	62	Swelling	Absent	248	Absent	–	–	Absent	Absent	Absent	
22	F	1.8	8	Erythema	Absent	92	Absent	–	–	Absent	Absent	Absent	
23	F	1.6	144	Swelling	Absent	170	Absent	–	–	Absent	Absent	Absent	
24	M	1.5	42	Erythema	Absent	79	Absent	–	–	Absent	Absent	Absent	
25	M	0.9	45	Fever	Absent	118	Absent	–	–	Absent	Absent	Absent	

CVC Central venous catheter, F Female, M Male, MRSA Methicillin-resistant *Staphylococcus aureus*

Nine patients underwent unplanned extraction of tunneled CVC due to complications within the subcutaneous tissue. Two of these patients experienced catheter fractures at the tunneled area. In those two cases, the times from catheter insertion to symptom onset were 3 and 45 days. The other seven patients experienced infections at the tunneled areas, with an average time from catheter insertion to symptom onset of 172.9 ± 217.7 (range, 14–611) days. The pathogens of infection were methicillin-resistant *Staphylococcus aureus* (two patients), *Staphylococcus aureus* (three patients), both *Pseudomonas aeruginosa* and *Serratia marcescens* (one patient), and *Enterococcus faecalis* (one patient). Of the nine patients, two were successfully treated with antibiotics, and unplanned extraction could therefore be avoided.

### Comparison of patient characteristics

Table 2 compares patient characteristics and summarizes sonographic findings between patients with and without unplanned CVC extraction. Significant differences were observed in the presence or absence of clinical diagnosis of infection and in the time from symptom onset to CVC extraction (presence or absence of infection with unplanned CVC extraction vs. presence or absence of infection without unplanned extraction = 7/2 vs. 2/14, P = 0.002; time from symptom onset to CVC extraction (days) with unplanned CVC extraction vs. those without = 3.4 ± 16.2 (0–47) vs. 168.9 ± 101.2 (4–415) days, P < 0.001). No significant differences were found between the two groups for patient characteristics such as sex, age, or time from catheter insertion to symptom onset (female/male with unplanned CVC extraction vs. those without = 4/5 vs. 10/6, respectively, P = 0.434; age of those with unplanned CVC extraction vs. those without = 5.5 ± 3.5 (0.3–10.9) vs. 6.6 ± 4.9 (0.4–15.0) years, respectively, P = 0.598; and time from catheter insertion to symptom onset in those with unplanned CVC extraction vs. those without = 139.8 ± 188.5 (3–611) vs. 74.5 ± 94.6 (1–389) days, respectively, P = 0.559).

**Table 2:** Comparison of patient characteristics and sonographic findings by presence or absence of unplanned extraction of tunneled central venous catheter

	Patients with unplanned CVC extraction (N = 9)	Patients without unplanned CVC extraction (N = 16)	P-value
Females/males	4/5	10/6	0.434
Age (years)	5.5 ± 3.5 (0.3–10.9)	6.6 ± 4.9 (0.4–15.0)	0.598
Time from catheter insertion to symptom onset (days)	139.8 ± 188.5 (3–611)	74.5 ± 94.6 (1–389)	0.559
Time from symptom onset to catheter removal (days)	13.4 ± 16.2 (0–47)	168.9 ± 101.2 (4–415)	< 0.001
Clinical diagnosis of infection at tunneled area (presence/absence)	7/2	2/14	0.002
Ultrasound findings			
High echogenicity around catheter within subcutaneous tissues (presence/absence)	7/2	6/10	0.097
Hypoechoic effusion around catheter within subcutaneous tissues (presence/absence)	9/0	3/13	< 0.001
Hyperechoic foci around catheter (presence/absence)	3/6	1/15	0.116

### Comparison of sonographic results between the patients with and without unplanned extraction of tunneled CVC

A significant difference between the patients with and without planned extraction was found in hypoechoic effusion around the catheter within the subcutaneous tissues (those with vs. without unplanned extraction = 9/0 vs. 3/13; P < 0.001). No significant differences were found in the presence/absence of high echogenicity (those with vs. without unplanned extraction = 7/2 vs. 6/10, P = 0.097) or hyperechoic foci around the catheter (those with vs. without unplanned extraction = 3/6 vs. 1/15, P = 0.116). However, all four patients with hyperechoic foci around the catheter also had complications at tunneled areas, such as infection or fracture.

### Time from symptom onset to catheter removal compared to the presence or absence of sonographic findings

The times from symptom onset to catheter removal compared to the presence or absence of sonographic findings (including hyperechoic area around catheter, fluid collection around catheter, and hyperechoic foci around catheter) are shown in Table 3. Significant differences in the times from symptom onset to catheter removal were observed in hypoechoic effusion and hyperechoic foci but not in high echogenicity (those with period with presence of hypoechoic effusion around catheters vs. those without = 44 ± 66 (0–231) vs. 176 ± 106 (4–415), P = 0.001; those with periods with a presence of hyperechoic foci around catheters vs. those without = 24 ± 39 (0–91) vs. 130 ± 112 (1–415), P = 0.025; those with periods with a presence of high echogenicity around catheters vs. those without = 82 ± 95 (0–287) vs. 146 ± 116 (4–415), P = 0.123).

**Table 3:** Comparison of time (days) from symptom onset to catheter removal by presence or absence of sonographic findings

	Time from onset of symptom to removal of catheter between the two groups (days)		P-value
	Presence of sonographic finding	Absence of sonographic finding	
High echogenicity around catheter within subcutaneous tissues (presence/absence = 13/12)	82 ± 95 (0–287)	146 ± 116 (4–415)	0.123
Hypoechoic effusion around catheter within subcutaneous tissues (presence/absence = 12/13)	44 ± 66 (0–231)	176 ± 106 (4–415)	0.001
Hyperechoic foci around catheter (presence/absence = 4/21)	24 ± 39 (0–91)	130 ± 112 (1–415)	0.025

## Discussion

Sonographic images of hypoechoic effusion around the catheter within subcutaneous tissues may indicate abscess formation or fluid leakage [8]. Since clinical evaluation is crucial to the decision of whether to extract the catheter, the sonographic findings of this study will be useful for determining the feasibility of CVC extraction. The CVC extracted after shorter periods had a greater tendency to have hypoechoic effusion and hyperechoic foci. Infection at those areas could prompt unplanned CVC extraction. However, because of venous access difficulty, two patients who were diagnosed with infection were treated with antibiotics and were therefore able to avoid unplanned extraction. Therefore, CVC management should be based on patient condition in addition to sonographic results.

In some patients, the time from symptom onset to CVC extraction was not necessarily affected by the presence or absence of infection. In most patients diagnosed with infection, CVC extraction was recommended. However, if antibiotic treatment was selected instead, extraction was delayed in order to evaluate the effects of the treatment. In one case, the symptoms diminished without specific treatment; however, the CVC was still extracted because the patient continued to have symptoms only 4 days prior to the planned extraction.

We observed two types of complications in our small cohort: catheter fracture and catheter infection (although most were infection). This trend is consistent with previous reports [17]. Catheter fracture management involves only catheter removal and replacement, whereas infection requires antibiotic therapy and may or may not require removal [2, 7, 10, 13, 17–19]. Therefore, carefully distinguishing between these complications is important. Hyperechoic foci indicate the presence of air bubbles, which may result from catheter leakage or infection [20, 21]. Even though only two patients in our study had fracture complications, both exhibited hyperechoic foci. Therefore, in patients with this finding and no suspected infection, further evaluation of the catheter using contrast media is recommended (Fig. 1). Furthermore, in these two patients, the time from catheter insertion to onset of fracture complications may have been shorter after surgical intervention than in patients with catheter infection. Various factors, such as frequency, power, and mechanical damage during catheter insertion, may also affect the incidence of catheter fracture. However, infection at the site of surgical intervention may occur days or weeks after surgery, which is consistent with our result [22, 23].

Inflammatory changes around the catheter may occur because of mechanical stimulation or infection and can be visualized as a highly echoic area around the catheter [24, 25]. In the two cases of fracture complications, this was observed both with and without unplanned CVC extraction. No significant difference was found in time from onset of symptoms to catheter removal.

Our study had some limitations. First, we included only 25 pediatric patients. Clinically, some patients were diagnosed with complications within the subcutaneous tissue and the tunneled areas without ultrasound examination. Second, we used two types of catheters in our study: Hickman and Broviac. These two types are similar, but the Hickman has double lumens and the Broviac has a single lumen. Although both types are widely used, the small number of patients in our cohort did not allow us to compare the incidence of complications between these two types of catheters. Moreover, various pathogens were associated with infectious complications in our study; however, we could not compare the differences between them. Therefore, further studies with a larger sample size, and in which all patients have the same types of tunneled catheters, are needed to validate our results.

## Conclusions

Sonographic findings of hypoechoic effusion around catheters within subcutaneous tissues indicate the necessity of tunneled CVC extraction. Furthermore, tunneled CVC that had hypoechoic effusion and hyperechoic foci around catheters in subcutaneous tissue tended to be extracted after shorter periods of time. This information will be useful in the management of pediatric patients receiving CVC.

## Data Availability

The authors confirm that the data supporting the findings of this study are available within the article in Table 1.
